# Exploring of two different equated instability resistance training programs on measure of physical fitness and lower limb asymmetry in pre-pubertal weightlifters

**DOI:** 10.1186/s13102-023-00652-0

**Published:** 2023-03-23

**Authors:** Raouf Hammami, Hadi Nobari, Werfelli Hanen, Javier Gene-Morales, Haithem Rebai, Juan C. Colado, Luca Paolo Ardigò

**Affiliations:** 1 Higher Institute of Sport and Physical Education of Ksar-Said, University of La Manouba, Tunis, Tunisia; 2 Research Laboratory Education, Motricity, Sport, and Health LR19JS01, Higher Institute of Sport and Physical Education of Sfax, Tunis, Tunisia; 3 Department of Exercise Physiology, Faculty of Educational Sciences and Psychology, University of Mohaghegh Ardabili, Ardabil, 56199-11367 Iran; 4 Faculty of Sport Sciences, University of Extremadura, Cáceres, 10003 Spain; 5 Prevention and Health in Exercise and Sport (PHES) research group, University of Valencia, Valencia, Spain; 6 Research Institute on Traffic and Road Safety (INTRAS), University of Valencia, Valencia, Spain; 7 Department of Teacher Education, NLA University College, Linstows Gate 3, Oslo, 0166 Norway

**Keywords:** Unstable surfaces, Adolescent athletes, Children training, Center of pressure, Hop tests

## Abstract

**Background:**

Although previous research in pediatric populations has reported performance enhancements following instability resistance training, the effects of different volumes on measures of balance, strength and power and lower limb asymmetry remain unclear.

**Objective:**

To compare the effect of two 8-week instability resistance training programs (IRT1: 2 sets x 8 repetitions, 20% one-repetition maximum (1RM); IRT2: 2 sets x 4 repetitions, 40% 1RM) in balance (center of pressure displacements), 1RM, single-leg jumps, and inter-limb asymmetry of prepubescent weightlifters.

**Methods:**

Thirty-two male athletes (10.94 ± 0.47 yrs, 17.05 ± 0.14 kg/m^2^, and 6.44 ± 0.93% bodyfat) voluntarily participated and combined IRT (front and back squat, lunge, and deadlift) with their usual weightlifting training. Nonparametric tests evaluated the differences between pre- and post-intervention.

**Results:**

Significant improvements were encountered in all the variables for both groups (effect sizes [ES] from 0.46 to 2.60), except the inter-limb asymmetry and the velocity of displacement of the center of pressure, which did not improve in IRT2. It is also worth highlighting that in the single-leg jumps and center of pressure displacements, whereas no significant differences were observed between IRT1 and IRT2 in the baseline, significant differences appeared in the follow-up measurements.

**Conclusion:**

IRT combined with weight-lifting training improves prepubescent weightlifters’ performance; therefore, strength and conditioning coaches may consider including unstable devices with low loads into an overall conditioning program and warm-ups for prepuberal male weightlifters to promote their physical fitness and potentially decrease inter-limb asymmetry.

**Trial registration:**

This study does not report results related to healthcare interventions using human participants; therefore, it was not prospectively registered.

## Background

Olympic weight-lifting consists of lifting the heaviest weight possible during two events, the snatch and clean and jerk. Both lifts require the ability to produce high levels of strength and power while maintaining balance [1, 2]. Given that Olympic weight-lifting is associated with high-velocity perturbations to trunk stability and maintenance of balance [1], weightlifters likely experience adaptations in acquiring and integrating sensory information and motor strategies for maintaining stability and balance. Current training facilities commonly use instability resistance training (IRT) [3, 4]. IRT involves exercises with body mass as resistance or external loads (e.g., dumbbells and/or barbells) performed on an unstable surface (e.g., Bosu, fitball, balance boards and/or foam platforms).

However, some controversy exists on the appropriateness of resistance exercises on unstable surfaces for athletes. Several authors reported that weight training on stable surfaces might produce a more efficient stimulus for stabilizing the trunk than specific callisthenic exercises [5] or the same movement on unstable surfaces [6]. Also, Chulvi-Medrano et al. [7] recommended not using irregular surfaces in training with loads above 70% of the one-repetition maximum (1RM). More recently, Behm et al. [8] summarized the effects of IRT across the lifespan and pointed out that a greater degree of instability provides tremendous stress and, thus, more significant opportunities for training adaptations of the neuromuscular and balance systems. In contrast, its use is only partially recommended [8]. However, these authors failed to identify studies examining IRT’s effects on children. Therefore, this training method’s effectiveness for optimal sports performance in the pediatric population has yet to be studied.

There is some controversy on the appropriateness of performing resistance exercises in children. There is a widely held inaccurate belief that strength training, when conducted during puberty and/or adolescence, can hamper one’s growth by damaging growth plates. Instead, strength training is safe and does not negatively impact the development and maturation of pre- and early-pubertal youth [9, 10]. Benefits include increased strength, speed, and power, improved body composition, stronger bones and reduced injury rates [11, 12].

When prescribing resistance exercise for youth, it is essential to consider program variables, e.g., intensity, repetitions, sets and training frequency [3, 13, 14]. These variables can all be modified depending on the subject’s characteristics to emphasize different adaptations [15, 16]. To the authors’ knowledge, there is no study available that examined the effects of various adjustments of program variables (i.e., percentage of repetition maximum and volume) in IRT on selected balance, muscle strength and power in young weightlifters who have less developed neuromuscular system than adults [17].

Therefore, this study aimed at verifying the effect of two IRT programs (low-series and high repetitions [IRT1] vs. moderate-series and low-repetitions [IRT2]) equated in terms of the degree of mechanical tension (i.e., a function of intensity (amount of load) and time under tension (duration of applied load); [18]) on muscle strength (1RM), power (single-leg-hop test and three-hop jump test), lower limb asymmetry (inter-limb performance imbalance) and postural sway (center of pressure surface area [CoP SA], lateral [CoP X], anteroposterior [CoP Y] displacement and center of pressure velocity [CoP V]) of pre-pubertal young weightlifters. Bearing in mind the principle of IRT specificity [13, 19] and that balance and coordination are not fully developed in young weightlifters [3, 17], we hypothesized that low-intensity high-volume (IRT1) would result in more significant improvements in selected balance variables, strength, and power performance in young weightlifters.

## Method

### Experimental approach to the problem

A mixed-factorial, within-and-between-factors interaction with a repeated measures design (two training methodologies [between-subject factor], baseline and follow-up measurements [within-subject factor]) was used to test the study hypotheses. Similar to other previously published studies [20, 21], a proper control group could not be incorporated as the two experimental groups were youth athletes and no comparable athletes available to provide similar baseline values.

### Participants

An a priori power analysis [22] of the required sample size suggested an n of 32 subjects to obtain a statistical power (1-ß) of 0.80, of 0.05 and effect size (f) of 0.26. Accordingly, 32 young male weightlifters between 10 and 12 years of age, who were members of the national Tunisian Weight-lifting Promotion Center (Kalaat el Andalous, Bizerte, Tunisia), volunteered to participate in this study. Athletes were assigned to either low-intensity high-volume (IRT1, n = 16) or moderate-intensity low-volume (IRT2, n = 16) instability resistance training. Groups were matched for age, maturation status and physical characteristics. All participants were from similar socioeconomic statuses and had the same daily school-training schedules. Since they lived in the same city, all participants’ environmental conditions for testing and training were identical. None were involved in after-school activities or formalized strength and conditioning training programs besides their weight-lifting preparation. No athletes had any history of musculoskeletal, neurological or orthopaedic disorder that might impair their ability to execute resistance or balance activity or to perform power, strength or balance tests. Further descriptive features of the sample are displayed in Table 1.

**Table 1:** Characteristics of the study participants

Characteristic	IRT1 group		IRT2 group		p-value
	M	SD	M	SD	
**Age (years)**	11.06	0.36	10.82	0.58	0.154
**Height (cm)**	151.06	6.12	147.50	8.12	0.172
**BM (kg)**	39.49	5.97	36.25	7.81	0.207
**BF %**	6.37	0.90	6.59	0.96	0.524
**PHV (years)**	-1.33	0.90	-1.85	0.83	0.099
**APHV (years)**	12.41	0.84	12.68	1.15	0.455

Values are presented as mean (M) and standard deviation (SD) and level of significance (p-value) of the comparison between groups. BM: body mass; BF%: body fat percentage; PHV: peak height velocity; APHV: predicted age at PHV. Previous research on the calculations of PHV and APHV can be consulted [23, 24]

To estimate the maturity status of participants, a maturity index (i.e., timing of maturation) was calculated [25]. This assessment is a non-invasive and practical method of predicting years from peak height velocity (PHV) as a measure of maturity offset using height and age as variables. The present study was conducted following the latest version of the Declaration of Helsinki and the protocol was fully approved by the Local Ethics Committee of the National Centre of Medicine and Science of Sports of Tunis (CNMSS-LR09SEP01) before the commencement of the procedure. Informed consent from parents/legal guardians for participants below 16 are involved in the study.

### Procedures

All the procedures were carried out during pre-season (September-October 2020) and lasted six days. The weekly weight-lifting training routine of the subjects comprised five training sessions per week (~ 90 min), with each session consisting of mainly technical-specific training (snatch and clean and jerk). Before the study’s commencement and initiation of testing, all athletes completed a two-week orientation period (3 sessions/week) to become familiar with the general environment, form and technique of each training program exercise and study test. During this time, the subjects received consistent instructions from certified strength and conditioning specialists. Each participant’s height and body mass were measured using a wall-mounted stadiometer and electronic scale. The same-trained investigator measured two skinfold thicknesses (triceps and subscapular) in triplicate. Measurements were made on the right-hand side of the body using a Harpenden calliper (Baty International, West Sussex, England). The body fat percentage was calculated using the equations of Slaughter et al. [23] for boys.

The performance-testing battery included assessments of postural sway variables relative to the center of pressure (CoP; mediolateral and anteroposterior displacement [CoP X and Y], surface area [CoP SA] and velocity [CoP V]), muscle strength (squat 1RM), power (three-hop and single-leg-hop test with dominant and non-dominant leg) and lower limb asymmetry (percentage of the difference between dominant and non-dominant leg in the single-leg-hop test). Each test consisted of three trials with a 60-s rest and 2 min between tests [24]. All the tests were conducted in a randomized counter-balanced order. Before familiarization and testing sessions, all participants completed a standardized warm-up protocol. A 3-minute rest was prescribed between the warm-up and the first test.

### Static balance

Static balance was evaluated as a crucial skill in pre-pubertal sport participation [26]. A force plate with three strain gauges (PostureWin, Techno Concept, Cereste, France; 40 Hz frequency, 12-bits A/D conversion) was used to measure the CoP displacements. The force plate was embedded in the surrounding floor. Participants were asked to stand as still as possible during testing with their arms comfortably placed downward at either side of the body, their bare feet separated by an angle of 30° and their heels placed 5 cm apart. To maintain the same foot position for the balance assessment, a plastic device was used to allow replication of the foot position [27]. Participants were asked to maintain balance on a firm surface, looking at a cross placed at eye level on a nearby wall (2 m distance). Each trial lasted 25.6 s. In this study, the centre of pressure displacement surface area (CoP SA), mediolateral displacement length (CoP X), anteroposterior displacement length (CoP Y) and velocity of displacement (CoP V) were selected as postural balance variables. CoP V indicates the total distance covered by the CoP divided by the duration of the sampled period, and CoP SA represents the area covered by the trajectory of the CoP [27]. For these variables, the lower the value, the better the postural control [28]. Excellent levels of reliability were observed (CoP SA: ICC = 0.873 and CV = 11.03%; CoP X: ICC = 0.855 and CV = 11.68%; CoP Y: ICC = 0.918 and CV = 8.30%; CoPV: ICC = 0.897 and CV = 8.16%).

### Dynamic strength

Lower-body dynamic strength was assessed with a 1RM parallel squat [29, 30]. Before attempting the 1RM, subjects performed three sub-maximal sets of 1–6 repetitions with light-to-moderate loads. Then, they performed a series of single repetitions with increasingly heavier loads. The weight increments depended on the effort required for the lift and became progressively smaller as the subject approached their 1RM. Failure was defined as a lift falling short of the full range of motion on at least two attempts, spaced at least 2 min apart. The 1RM was determined within 6 to 8 tries. Throughout all testing procedures, an instructor-to-subject ratio of 1:1 was maintained. Uniform verbal encouragement was offered to all subjects. The test showed excellent test-retest reliability (ICC = 0.801 and CV = 6.43%).

### Horizontal jump

Due to the importance of horizontal force and power in explosive tasks with youth weightlifters [31], two horizontal jumps were used in this study: three-hop (3 H) and single-leg hop (SLH) tests with the dominant and non-dominant leg.

In the 3 H test (for distance), subjects began standing on the dominant leg, with hands on their hips and toes behind the starting line. They were instructed to perform three maximal hops forward (landing on the same leg throughout) to minimize ground contact times after the first and second hops. When landing from the final hop, subjects were required to ‘stick’ the landing and hold for 2 s. Failure to stick the final landing resulted in a void trial and the jump being retaken after a 60-second rest. The distance from the starting line to the landing position of the subjects’ heels was then measured and recorded to the nearest centimeter [32] using a standard measuring tape fixed to the floor (used for all hop tests). Excellent reliability values were observed (ICC = 0.963 and CV = 1.72%).

As previously described [33], in the SLH test (for distance), subjects began standing on the designated testing leg with their hands on hips and their toes behind the starting line. Subjects had to hop as far forward as possible and land on the same leg. Upon landing, participants were required to hold their position for 2 s. Failure to stick the landing resulted in a void trial and the jump being retaken after 60 s. The distance from the starting line to the point where the participant’s landing heel hit in the final position was recorded to the nearest centimeter. Excellent test-retest reliability was perfect for both SLH tests with the dominant (ICC = 0.939 and CV = 3.19%) and the non-dominant leg (ICC = 0.905 and CV = 4.45%) in youth athletes.

Bilateral inter-limb asymmetry was calculated with the outcomes of the SLH using the formula (more muscular leg–weaker leg)/stronger leg×100 [33]. A negative sign (-) was arbitrarily assigned when the left leg was the stronger one, and a positive sign (+) was used when the right leg was the stronger one [33]. Test-retest reliability was high for the lower limb asymmetry (ICC = 0.648 and CV = 34.21%).

### Training program

Both IRT programs were based on recommendations and training guidelines for the pediatric population [34, 35] and integrated into the regular training routine of the subjects, maintaining overall training volume. Training programs were performed twice weekly for eight weeks on alternate days to provide a sufficient resting period between sessions. Each session lasted 60 min and started with a standardized 15-minute warm-up. The standardized warm-up included jogging, dynamic stretching and calisthenics exercises (multi-planar lunges, inchworms and spiderman planks and walks) and range of motion preparation [36] before progressing into practice jumps and sprints at 60, 80 and 100% of perceived maximum effort. During the orientation weeks and at the beginning of each training session, preparatory exercises (e.g., fundamental weight-lifting exercises specific to their training program) were performed by both groups. Each training session ended with five minutes of cool-down activities, including dynamic stretching.

The participants were randomly allocated into one of two groups. Every participant was identified by an identification number entered into an online randomizer (https://www.randomizer.org/) which arranged the participants into two groups. Participants in both IRT1 and IRT2 executed strengthening exercises on unstable surfaces frequently used for athletic training and rehabilitation (e.g., Airex Balance Beam, Airex Balance Pad, Thera-Band Stability Trainer and Togu Aero Step). The low-intensity high-volume instability resistance training program (IRT1) consisted of 2 sets × 8 repetitions at 20%1RM, whereas the moderate-intensity low-volume program (IRT2) consisted of 2 sets × 4 repetitions at 40%1RM. Both experimental groups used the same instability throughout the training program. Subjects of both training groups were requested to execute the concentric phase of the exercises at maximum speed. To equate the total training load, volume (duration of applied load) and intensity (including weight and velocity of execution) prescribed each week were identical (i.e., IRT1: 4 exercises × 2 sets × 8 repetitions at 20%1RM; IRT2: 4 exercises × 2 sets × 4 repetitions at 40%1RM). This measure is also reported in the literature as the degree of mechanical tension [18]. The four exercises performed were the back and front half-squat [29, 37], forward lunge and deadlift [15]. According to previous research [38], rest intervals of 3 min between sets were ensured in both training methods. The time under tension of each training program was equated for both groups, usually between 30 and 60 s, depending on how many repetitions and sets the two training groups performed. Of note, the rest of the training program conducted with the number of sets (3 min) and repeat (1 min) and between training sessions (48 h) was kept the same to avoid any interference and was similar for both groups.

### Statistical analysis

All the statistical analyses were performed using the IBM SPSS version 28.0 (Armonk, NY, IBM Corp). After basic data curation, descriptive statistical analyses were carried out. Data were organized into two groups (IRT1 and IRT2) with two moments of measurement (pre-intervention and post-intervention) to be further analyzed with the inferential analysis. The normality of the data distribution was tested with the Shapiro-Wilk procedure. Most variables showed a non-Gaussian distribution. However, most complied with homogeneity of variance assumption, only the squat 1RM in the preintervention measure showed nonhomogeneous variances. Considering its robustness [39], parametric analysis of variance (ANOVA) was chosen to evaluate differences between the dependent variables. A cut-off criterion of p ≤ .05 was uniformly established to identify statistical significance.

Therefore, a mixed-model ANOVA was conducted with the time (preintervention and postintervention) and the group (IRT1 and IRT2) as the within- and between-subject factors, respectively. The effect size was reported as partial eta squared (ηp^2^), being interpreted as small (0.01<ƞp²<0.06), moderate (0.06≤ƞp²≤0.14) and large (> 0.14) [40]. Posthoc comparisons with Bonferroni corrections were conducted in all cases. The effect size for posthoc comparisons was calculated as Cohen’s d with Hedges corrections. This value is reported as unbiased Cohen’s d (dunb) [41], with dunb < 0.50 constituting a small effect, 0.50 ≤ dunb ≤ 0.79 moderate and dunb ≥ 0.80 a large effect [40].

## Results

All the subjects participating in the study completed the intervention. The ANOVA showed a significant effect of time in all the study variables (p values between < 0.001 and 0.005; ηp^2^ between 0.233 and 0.820). On the other hand, the interaction group*time resulted in nonsignificant in all the study variables (all p > .050). The descriptive and inferential posthoc outcomes obtained for strength (1RM), power (jump tests), and balance (CoP displacement) tests can be found in Table 2.

**Table 2:** Pre-and-post-intervention values of IRT1 (low-intensity high-volume instability resistance training [2 sets × 8 repetitions 20%1RM], n = 16) and IRT2 (moderate-intensity low-volume instability resistance training [2 sets × 4 repetitions 40%1RM], n = 16) in strength, power, and balance tests

Variable	Group	Baseline			Follow-up		Δ%	Sig.	ES
		**M**	**SD**		**M**	**SD**			
**Strength and power variables**									
1RM (kg)	IRT1	36.06*		4.91	45.94*	3.66	27.40	**< 0.001**	**2.24**
	IRT2	30.69		2.94	40.75	5.00	32.78	**< 0.001**	**2.39**
SLH (Dominant; cm)	IRT1	170.63		19.74	182.19*	15.81	6.77	**0.003**	**0.63**
	IRT2	158.44		21.58	167.81	17.41	5.91	**0.015**	**0.47**
SLH (Non-dominant; cm)	IRT1	145.00		22.21	162.81	17.22	12.28	**< 0.001**	**0.87**
	IRT2	138.13		14.24	151.88	15.80	9.95	**< 0.001**	**0.89**
3 H (cm)	IRT1	449.38		30.87	516.25*	42.56	14.88	**< 0.001**	**1.75**
	IRT2	423.13		39.79	469.69	64.56	11.00	**0.001**	**0.85**
Lower inter-limb asymmetry	IRT1	15.09		7.31	10.66	5.16	-29.36	**0.015**	**0.68**
	IRT2	12.29		6.20	9.41	4.20	-23.43	0.103	0.53
**Balance variables**									
CoP SA (mm^2^)	IRT1	1940.17	718.80		548.21*	164.47	-71.74	**< 0.001**	**2.60**
	IRT2	2158.47	799.11		692.80	216.02	-67.90	**< 0.001**	**2.44**
CoP X (mm)	IRT1	2110.29	660.59		1687.12	608.50	-20.05	**0.019**	**0.65**
	IRT2	2134.54	668.69		1690.68	613.69	-20.79	**0.014**	**0.67**
CoP Y (mm)	IRT1	2110.78	540.92		1728.89	477.35	-18.09	**0.002**	**0.73**
	IRT2	2247.15	706.96		1936.52	603.05	-13.82	**0.008**	**0.46**
CoP V (mm/s)	IRT1	63.61	16.74		50.76	15.20	-20.20	**0.005**	**0.78**
	IRT2	66.37	18.61		60.34	13.27	-9.09	0.166	0.36

Notes. * Statistically significant difference between baseline or follow-up measurement groups. 1RM: one repetition maximum; SLH Dominant: single leg hop test with the dominant leg; SLH Non-Dominant: single leg hop test with the non-dominant leg; 3 H: triple hop test; CoP SA: surface area of the center of pressure oscillation; CoP X: lateral displacement of the center of pressure oscillation; CoP Y: anteroposterior displacement of the center of pressure oscillation; CoP V: center of pressure oscillation velocity; M: mean; SD: standard deviation; Sig: p-value of the significance of the posthoc test (pre-post); ES: Effect size (Cohen’s d)

The most noteworthy findings regarding the within-subject comparisons were that both groups significantly improved almost all the assessed variables (effect sizes [ES] from 0.46 to 2.60). Only the interlimb asymmetry (p = .103) and CoP V (p = .166) of IRT2 presented nonsignificant variaitons.

Regarding betweengroup comparisons, it is worth highlighting that significant differences in the baseline values appeared in the squat 1RM (p < .001) and 3 H (p = .046). Significant differences emerged in the follow-up measurements of the squat 1RM (p = .002), SLH with the dominant leg (p = .021), 3 H (p = .022), and CoP SA (p = .041). Nonsignificant differences were observed between IRT1 and IRT2 in the rest of the dependent variables (p > .05).

## Discussion

To the authors’ knowledge, this is the first study that investigated the effects of two different instability training programs on the athletic performance of pre-pubertal weightlifters. The most relevant finding was that eight weeks of IRT and regular weight-lifting training effectively enhanced specific parameters of balance, muscle strength, and power in prepubescent weightlifters. Most athletic performance measures (i.e., 1RM, 3 H, SLH, CoP SA, CoP X, and, CoP Y) were significantly enhanced after the 8-week training period for both groups (see Table 2). More specifically, IRT1 (2 sets × 8 repetitions 20%1RM) improved all the variables analyzed, and IRT2 (2 sets × 4 repetitions at 40%1RM) did not enhance inter-limb asymmetry (as measured by the performance differences between legs in a horizontal jump) and CoP displacement velocity as a measure of balance. Considering that balance and coordination are not fully developed in children [34], the results of the present study should therefore be translated into regular weight-lifting training to benefit from success in training and competition. Thus, IRT can be considered a means to improve performance in young weightlifters.

Considering the nonsignificant effect of the interaction time*group, it could not be suggested that any IRT programs provided athletes with more excellent balance, muscle strength, and power improvements, which only partially supports our initial hypothesis. Amar et al. [42] showed that the change in the standing stork test among a IRT group was substantially greater than that among a traiditional resistance training group (p = .007) but did not significantly differ from that among a plyometric training group (p = .270) in young adults. Accordingly, both IRT programs seem to adequately stress the neuromuscular system and induce apppropriate neuromuscular adaptation [42], in addition to causing high muscle activation, especially antagonist and co-contractions [43]. On the other hand, using a lower load under the same instability conditions allows for greater control, and force production and, therefore, could entail more excellent adaptations [11, 13]. It has been demonstrated that co-contractile (antagonist) activity increases on unstable surfaces with greater loads [19, 44]. In this sense, caution should be applied since IRT with low repetitions and moderate load (40%1RM) may have promoted higher co-contractions with shorter latency periods to protect the joints [19, 44]. The role of these co-contractions is to control limb position, increase joint stiffness and provide stability [34, 45] and balance performance [34]. Therefore, these co-contractions do not increase the force production in the direction of the exercise and could limit the performance. More specifically, a mean force deficit of 29% with unstable surfaces compared with similar activities on a stable surface has been reported in the literature [13]. Several researchers have reported increases in muscle activation of both the trunk and limbs [43] and rate of perceived exertion [46] in IRT compared to training on stable surfaces.

In addition, IRT1 protocol induced similar performance improvements in strength and power performance measures compared to IRT2 in prepuberal male weightlifters. Cowley et al. [47] reported an increase in 1RM strength by 15% and work capacity by 16% following a low load of IRT in young adults. Given that prepuberal athletes’ hormonal situation (lack of circulating anabolic hormones) does most likely not allow muscle hypertrophy, we speculated that neural factors caused the observed marked improvements in strength and power performance of both groups in terms of increased motor unit recruitment (i.e., intra-muscular coordination) and better synergistic and less antagonistic muscle activation strategies (i.e., inter-muscular coordination) [48].

Despite the currently recognized critical relevance of between-limb imbalance for risk of injury and performance [49, 50], as far as we know, this is the first study to assess the impact of IRT on lower-limb asymmetries in prepuberal weightlifters. In this regard, it is worth highlighting that, while a nonsignificant time*group interaction was observed, only the low-intensity high-volume IRT (IRT1) obtained a significant change in the inter-limb asymmetry (ES = 0.68; p = .015), and IRT2 showed nonsignificant differences (ES = 0.53; p = .103). Pardos-Mainer et al. [51] reported that a neuromuscular training program was effective for speed (ES: -1.30 to -1.16) and CoD tests (ES: − 0.62 to − 0.61) but not in jumping (ES: − 0.09 to 0.28) and inter-limb asymmetries tests (ES: − 0.13 to 0.57) in adolescent female soccer players. The significant reduction in lower-limb asymmetry observed was attained through low-load instability exercises that required a high level of joint control on both the sagittal and frontal planes and tasks that used either body weight or weights. Indeed, unstable surfaces can enhance intermuscular coordination between agonist and antagonist muscles, permitting improved joint position control and reduced joint stiffness [13]. Furthermore, controlling a heavy load during IRT exercises forces the participants to distribute load uniformly between the two limbs: only in this way can subjects correctly perform the tasks. This component can reduce the lower-limb stabilisation time following a flight phase [52] and thus reduce the lower limb asymmetry in the study participants.

A possible limitation of the present study is that other parameters of exercise intensity (e.g., session rating of perceived exertion and velocity of execution) could have been included. Using such a non-invasive method to quantify the internal training load in each training session could be helpful in future study designs. Also, we did not include a control group in the current study that did the same training in a stable condition. Consequently, the present study’s outcomes must be interpreted with caution. In addition, since the athletes seem to have been completing their weight-lifting training, as usual, it cannot be discounted that their regular activity was responsible for at least some of the effect sizes observed. In addition, analysis asymmetry was calculated using the group mean value, which showed the variable nature of asymmetry, as represented by the large standard deviation. As previously suggested, monitoring changes in asymmetry should be done on an individual basis.

Furthermore, prepuberal weightlifters have particular characteristics, and our results cannot be directly extrapolated to other sports. In this way, further studies should be done considering maturity offset [25] to determine the possible influence of maturation on performance adaptations. Moreover, given that the acute effects of strength exercises are transient, we had to focus not only on the back squat test but also on selected other outcome strength measures (i.e., front squat). Future studies should, therefore, examine the effects of instability strength training on the measurement of back and front squat performance. In addition, muscle strength performance was not tested under sport-specific conditions in this study which may have prevented observing larger effects. Future studies should include balance tests during the performance of weight-lifting exercises. Athletes could be tested while performing the snatch, clean and jerk. Finally, the maturity of offset (i.e., PHV) in the athlete’s pre and post-training was not controlled. Therefore, future studies should be considered in this way. Since the current study’s findings are novel, more research is needed to reach safe conclusions regarding the effects of unstable surfaces on balance, muscle strength, and power in youth athletes.

## Practical applications

In summary, we could confirm that instability resistance training in combination with joint weight-lifting exercises does not interfere with improving the athletes’ muscle strength, power and balance development. This finding adds to previous research, which recommends using IRT with no load to enhance adolescent athletes’ strength, power, and balance and suggests that low-to-moderate loads (20–40%) can be used in instability training for such purpose. More specifically, the program with lower loads and higher volume (IRT1) provided similar results to those with moderate loads and lower volume (IRT2). These findings imply that pediatric strength and conditioning coaches may consider including unstable devices with low-to-moderate loads into an overall conditioning program and warm-ups for prepuberal male weightlifters to promote their physical fitness and potentially decrease inter-limb asymmetry. However, such a conclusion could be warranted if there was a control group that underwent the same program in a stable condition, since the athletes were also completing their standard weight-lifting training, the effect of this ‘normal’ resistance training (i.e., weight-lifting training) on the outcomes presented should be interpreted with caution.

## Data Availability

The data presented in this study are available on request from the corresponding author.

## Abbreviations

BM: Body mass
BF%: Body fat percentage
PHV: Peak height velocity
APHV: Predicted age at PHV. Previous research on the calculations of PHV and APHV
IRT1: Instability resistance training 1
IRT2: Instability resistance training 2
CoP SA: Center of pressor surface area
CoP V: Center of presser velocity
CoP X: Mediolateral displacement of the center of presser
CoP Y: Anterior displacement of the center of presser
ICC: Intraclass coefession of correlation
CV: Coefficient of variation
SEM: Standard error of measurement
ES: Effect size
